# Integrative genomic and transcriptomic dissection of salt tolerance for *Japonica* rice improvement

**DOI:** 10.3389/fpls.2025.1751273

**Published:** 2026-01-20

**Authors:** Jingli Gao, Tae-Heon Kim, Dong-Hyun Baek, Chang-Ju Lee, Woo-Geun Park, Sang Dae Yun, Suk-Man Kim

**Affiliations:** 1 Department of Crop Science, Kyungpook National University, Sangju, Republic of Korea; 2 Institute of Agricultural Science and Technology, Kyungpook National University, Daegu, Republic of Korea; 3 School of Applied Biosciences, Kyungpook National University, Daegu, Republic of Korea

**Keywords:** GWAS, marker-assisted breeding, QTL, rice, salt tolerance, transcriptomics

## Abstract

Soil salinity is a major abiotic stress limiting rice productivity, particularly in coastal and irrigated regions. *Japonica* rice, widely cultivated in temperate regions, is moderately sensitive to salt stress, especially during the seedling stage. To accelerate salt-tolerance improvement in *Japonica* backgrounds, we conducted large-scale multi-environment phenotyping of 225 diverse rice cultivars under controlled salt stress (0.7% NaCl) across two seasons (spring and summer 2024), followed by genome-wide association study (GWAS) and transcriptome profiling. Best linear unbiased predictors (BLUPs) derived from linear mixed-effects models effectively corrected seasonal, spatial, and environmental variations, revealing substantial genotypic differences in seedling-stage salt tolerance. GWAS identified five novel QTLs (*qSES3*, *qSES5*, *qSES6*, *qSES7*, and *qSES9*), with *qSES6* and *qSES7* exhibiting strong synergistic effects. The transcriptome analysis of the highly tolerant cultivar ‘IR73571-3B-11-3-K2’ identified 834 DEGs, revealing enriched stress-responsive activities, such as oxidoreductase activity and phenylpropanoid biosynthesis. Integrating the GWAS and transcriptomic data highlighted Os07g0635500 (cytochrome P450) as a key candidate gene, and the haplotype analysis identifying two haplotypes, with Haplotype 2 conferring superior tolerance. However, favorable QTLs and haplotypes were predominantly found in Tongil-type cultivars, suggesting limited representation in *Japonica* cultivars. Therefore, targeted introgression and marker-assisted selection will be required to transfer salt tolerance traits into *Japonica* cultivars. Overall, this study dissected salt stress responses in rice and providing a multidimensional resource and practical insights for the molecular breeding of salt-tolerant *Japonica* rice.

## Introduction

Abiotic stressors, including drought, salinity, extreme temperatures, and nutrient imbalances, significantly diminish global crop production, reducing yield and quality across agricultural systems (Zhu, 2016; Boyer et al., 2013). Soil salinity affects an estimated 424–833 million hectares, representing ~20% of irrigated agricultural land worldwide (FAO, 2021). Salinity induces osmotic stress, ion toxicity, and oxidative damage, impairing seed germination and plant development and productivity (Munns and Tester, 2008; Yang and Guo, 2018). Climate change exacerbates the effects of salinization, particularly in coastal and arid regions, through rising sea levels and erratic rainfall (Negacz et al., 2022). Addressing salinity’s effects on major crops will be critical to ensuring food security for a global population projected to reach 9.7 billion by 2050 (Vollset et al., 2020).

Rice (*Oryza sativa* L.), a staple food for over 3.5 billion people, provides more than 50% of the caloric intake in Asia, making it pivotal for global food security (Wing et al., 2018). In addition to its agronomic importance, rice serves as a model crop species due to its compact genome (~389 Mb), extensive genetic diversity, and the comprehensive resources available for genomic research (International Rice Genome Sequencing Project & Sasaki, 2005). These attributes have enabled the detailed dissection of the genetic basis of complex traits, including abiotic stress tolerance.

However, salinity remains one of the most serious abiotic stressors limiting rice productivity. Rice is moderately sensitive to salt stress, particularly during the seedling and reproductive stages, when high salt concentrations disrupt ion homeostasis, reduce photosynthetic efficiency, and induce oxidative stress, ultimately leading to substantial yield losses (Gregoria et al., 1997; Zeng et al., 2002). Because rice can be readily cultivated in hydroponic systems, it also serves as a convenient experimental model for evaluating salinity tolerance under controlled conditions. Therefore, the development of salt-tolerant rice cultivars is essential to sustain rice production in salt-affected regions (Ali & Yun, 2017).

Traditional breeding has produced salt-tolerant cultivars, such as ‘Pokkali’ and ‘Nona Bokra’, which have been deployed in saline regions (Gregorio et al., 2002), and linkage mapping using these genetic resources has identified key quantitative trait loci (QTLs) associated with salinity tolerance, including *Saltol* (Thomson et al., 2010), *qSKC1* (Lin et al., 2004), and *qST1*/*qST3* (Takehisa et al., 2004). The landmark gene *OsHKT1;5*, a high-affinity K^+^ transporter that selectively unloads Na^+^ from the xylem to maintain shoot Na^+^/K^+^ homeostasis and prevent ion toxicity, was identified in *Saltol* and *qSKC1* (Ren et al., 2005; Platten et al., 2006). While QTL mapping has provided important insights, its resolution is limited by the effects of biparental populations and narrow allelic diversity. To overcome such constraints, genome-wide association studies (GWAS), which leverage the diversity of natural populations to capture broader genetic variation and achieve higher mapping resolution, have emerged as a powerful complementary approach. Recent studies have further elucidated these mechanisms; for instance, genome-wide association studies have identified *OsWRKY53* as a key transcription factor regulating salt tolerance by modulating downstream genes involved in ROS detoxification and ion transport in rice landraces (Yu et al., 2023). Additionally, transcriptome profiling, which reveals differentially expressed genes (DEGs) under changing conditions, complements genomic approaches by offering critical insights into molecular responses to stressors. Integrating transcriptomic data with GWAS data facilitates the identification of candidate genes and regulatory networks, bridging genetic associations with physiological mechanisms (Ren et al., 2023; Lv et al., 2022).

Salt tolerance in rice is a complex quantitative trait influenced by multiple loci and multiple environmental factors, such as soil salinity, temperature, and moisture (Flowers, 2004). Multi-environment phenotyping is essential to capturing genotype-by-environment (G×E) interactions and ensuring robust GWAS results (Spindel et al., 2015). Best linear unbiased predictors (BLUPs), derived from linear mixed-effects models (LMMs), are used in GWASs to adjust phenotypic data for non-genetic variance, reducing residual error and enhancing statistical power (Holland and Piepho, 2024). By accommodating complex experimental designs, BLUPs enable the precise estimation of genetic effects and have improved the detection of salt tolerance loci across diverse rice populations in a previous study (Nayyeripasand et al., 2021).

Despite these advances, significant research gaps remain. Most identified salt tolerance QTLs and favorable haplotypes are enriched in *Indica* or Indica-derived (Tongil-type) cultivars, with limited representation in *Japonica* rice, which is widely grown in temperate regions and shows moderate salt sensitivity, especially at the seedling stage (Lee et al., 2003).

To address the complex genetic architecture of salt tolerance in rice, this study establishes an integrative framework combining multi-season phenotyping, genome-wide association analysis, and transcriptomic profiling. Specifically, we aimed to (1) conduct multi-season phenotyping to accurately evaluate seedling-stage salt tolerance and identify tolerant rice cultivars. (2) perform GWASs to detect novel QTLs associated with salt tolerance; (3) integrate transcriptomic data to identify key candidate genes and validate them against GWAS signals; and (4) provide multidimensional insights from phenotypic, genotypic, and expression-level data to facilitate the molecular breeding of *Japonica* salt-tolerant rice.

## Materials and methods

### Culture conditions and salt tolerance assays at the seedling stage

**Figure 1:**
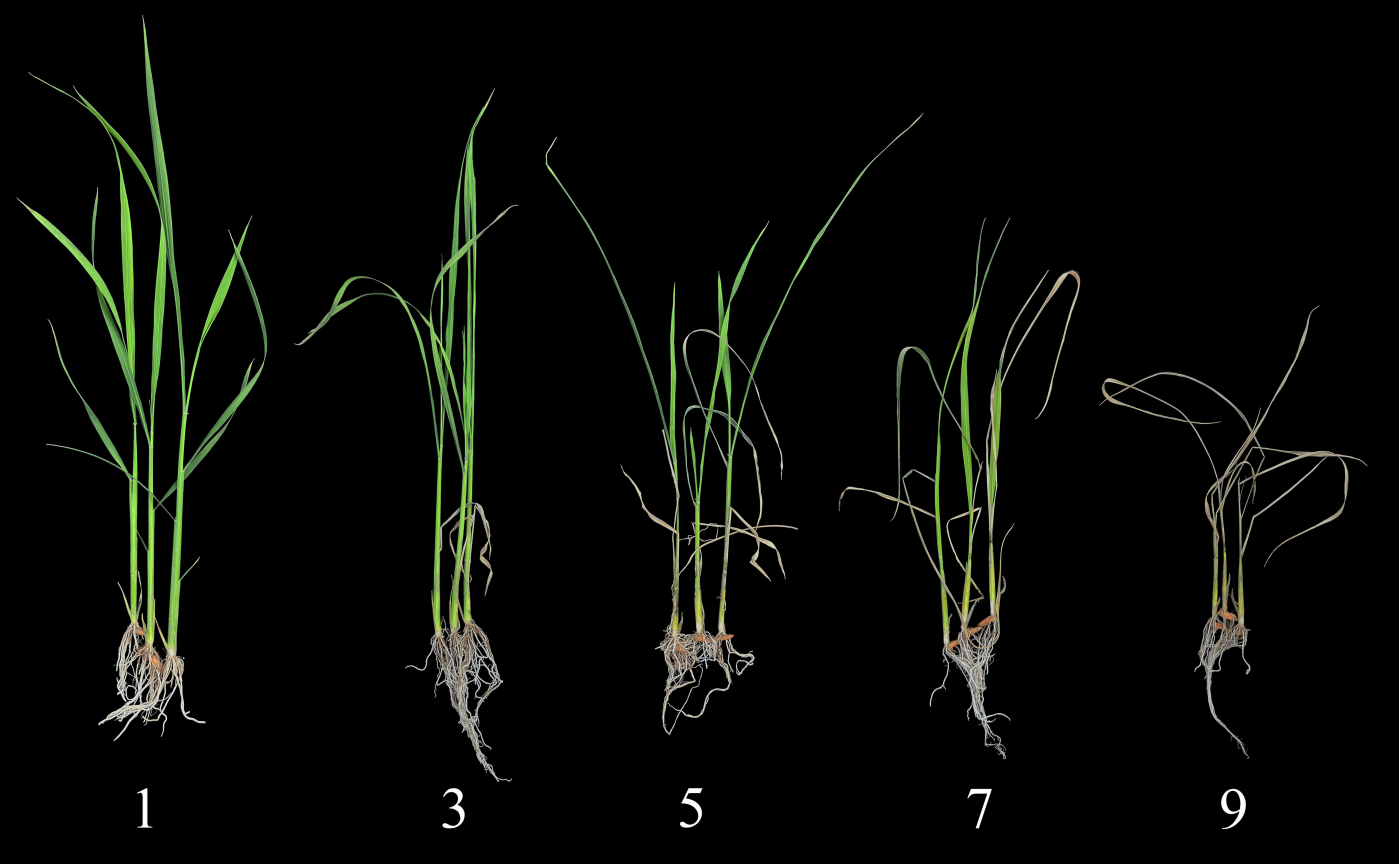
Example plants representing different scores of the visual salt injury evaluation performed at the seedling stage.

To break seed dormancy, dry seeds were incubated at 55 °C for 7 days. Seeds were then surface-sterilized and incubated at 30 °C for 72 hours to promote pre-germination. Uniformly germinated seeds were selected and sown in soil-filled seedling trays. Seedlings were initially irrigated with tap water. To assess salt tolerance, when seedlings reached the three-leaf stage (approximately 10 days post-sowing), salt stress was induced by submerging the trays in 0.7% (w/v) NaCl (approximately 120 mM) solution. This salt was selected because it provides discrimination between tolerant (‘Pokkali’) and susceptible (‘IR29’) genotypes, as established in previous screening studies (Gregoria et al., 1997). Prior to the salt stress treatment, abnormal or morphologically aberrant seedlings were removed to ensure uniformity. Individuals of the salt-sensitive rice cultivar ‘IR29’ were included in each tray as a susceptible control. Seedlings were visually evaluated for salt injury symptoms using a modified Standard Evaluation System (SES) for Rice (IRRI, 2013) when nearly all ‘IR29’ plants scored as 9 (**Figure 1**), typically 7 days after salt stress initiation.

Salt tolerance was assessed in 225 rice cultivars (**Supplementary Table S1**) over two growing seasons: spring (April–June) and summer (July–August) in 2024. A randomized complete block design was employed with seedling tray as the blocking factor to minimize spatial heterogeneity. Each tray was divided into forty 7 × 5 cm blocks (4 rows × 10 columns). Each cultivar was randomly allocated to one block in each of three independent trays. Each block contained 12 plants grown under standardized environmental and management conditions.

### Phenotype adjustment and linear mixed model analysis

To quantify phenotypic variation while accounting for the experimental structure and biological variability, a linear mixed-effects model was fitted using the “lmer()” function in the lme4 package of R (version 4.4.1). The model was defined as


$$y_{ijklm}\;=\;\mu \;+\;s_{i}\;+\;v_{j}\;+\;b_{k}\;+\;{\left(vb\right)}_{jk}\;+\;p_{m}\;+\;\epsilon_{ijklm}$$

where *y_ijklm_* represents the observed phenotypic score of the *i*-th season, *j*-th cultivar, *k*-th block, *l*-th replication, and *m*-th plant; *μ* is the overall mean; *s_i_* is the fixed effect of the season; $v_{j}\;\sim \;N(0,\;\sigma_{v}^{2})$ is the random effect of the cultivar; $b_{k}\;\sim \;N(0,\sigma_{b}^{2})$ is the random effect of the block; ${\left(vb\right)}_{jk}\;\sim \;N(0,\;\sigma_{vb}^{2})$ is the random interaction between cultivar and block; and $p_{m}\;\sim \;N(0,\;\sigma_{p}^{2})$ is the random effect at the individual plant level. The residual error term $\epsilon_{ijklm}\;\sim \;N(0,\sigma^{2})$ captures unexplained variability.

Season was modeled as a fixed effect to account for temporal environmental differences, while cultivar, block, the cultivar-by-block interaction, and plant ID were treated as random effects to capture the genetic and environmental variance components within the hierarchical experimental design. Best linear unbiased predictors (BLUPs) derived from the LMM were used as adjusted phenotypic values for subsequent analyses, including the GWAS.

### Genotypic data quality and population structure analyses

A total of 225 rice cultivars were genotyped. First, 198 cultivars were genotyped with the KNU Axiom Oryza 580K SNP array (581,006 markers; Kim K. W. et al., 2022). Genomic DNA was extracted from young leaves using the CTAB method. DNA quality and concentration were assessed using a NanoDrop ND-1000 spectrophotometer (Thermo Fisher Scientific, USA). Samples with ≥ 50 ng/μL, A260/A280 = 1.8–2.0, and A260/A230 > 2.0 were used. 200ng of DNA per sample were processed on the Affymetrix GeneTitan^®^ system following the AxiomTM 2.0 Assay protocol. Genotype calling and initial QC were performed using Axiom Analysis Suite v4.2. Samples with DQC < 0.82 or call rate < 97% were excluded. SNPs were further filtered using the SNPolisher R package (v1.8.0); only PolyHighResolution, NoMinorHom, and Off-Target Variant SNPs with call rate ≥ 97%, FLD ≥ 3.6, HetSO ≥ −0.1, and HomRO ≥ 0.3 were retained.

For the remaining 27 cultivars, raw whole-genome resequencing data were retrieved from the NCBI Sequence Read Archive (SRA accession numbers listed in **Supplementary Table S1**). Fastq files were obtained using prefetch and fasterq-dump from SRA Toolkit v3.0.0. Reads were aligned to the IRGSP-1.0 reference genome using BWA-MEM v0.7.17 with default parameters. BAM files were sorted and indexed using SAMtools v1.19. To ensure consistency with the Axiom array data, genotypes were called only at the exact 581,006 SNP positions present on the KNU Axiom Oryza 580K array. This was achieved using bcftools mpileup (-a FORMAT/AD,FORMAT/DP) followed by bcftools call restricted to array sites. The resulting VCF files for the 27 resequenced cultivars were then merged with the 198 Axiom array genotypes using PLINK v1.9. After removing SNPs with > 0.5% missing data or MAF < 0.05 across all 225 cultivars, a final set of 115,804 high-quality SNPs was retained for population structure analysis and GWAS.

Population structure was inferred using STRUCTURE v2.3.4, and a neighbor-joining tree and kinship matrix were constructed using TASSEL 5.0 and the VanRaden method in *GAPIT*, respectively. A principal component analysis (PCA) and per-chromosome LD analysis (based on *R*² and using a 50-marker window) were performed in TASSEL. The LD decay distance was estimated by fitting a nonlinear model following Otyama et al. (2019) with an *R*² threshold of 0.2, and defining the half-decay distance as the point at which *R*² equals 50% of its initial value (Huang et al., 2010).

### GWAS and QTL identification

The FarmCPU model was implemented to detect statistically significant marker-trait associations using the *GAPIT* package in R (version 4.4.1). The parameter of the PCA, total = 2, was applied to infer the population structure. FarmCPU was selected because it integrates fixed-effect and random-effect models to reduce false positives arising from population structure while maintaining high statistical power. Prior to analysis, the combined SNPs dataset was filtered using PLINK v1.9beta (Slifer, 2018) to ensure data quality. Variants with a missing rate > 0.005 or a minor allele frequency (MAF) < 0.05 were excluded. After quality control, 115,804 high-confidence SNPs were retained. Genome-wide significance thresholds were determined using the Bonferroni correction (*α* = 0.05), with *N* in the Bonferroni equation representing the total number of SNPs tested (115,804 in this study). Accordingly, the threshold was set at −log_10_(*P*) > 6.36.

For each significant SNP, a QTL region was defined as a 200-kb interval (100 kb upstream and downstream of the lead SNP) based on the genome-wide linkage disequilibrium (LD) decay distance estimated in this population (62–240 kb; **Supplementary Figure S2**). All annotated genes located within these 200-kb regions were extracted from the rice annotation of the IRGSP-1.0 genome assembly using the Rice Annotation Project database (https://rapdb.dna.affrc.go.jp/).

### Transcriptome analysis

One representative salt-tolerant cultivar, ‘IR73571-3B-11-3-K2’, was selected for a transcriptome analysis. Seedlings of ‘IR73571-3B-11-3-K2’ were treated following the method described above for salt-tolerance evaluation assays. Leaves were collected from each sample plant one day after the salt stress treatment, with water-treated seedlings serving as the control. Transcriptome sequencing was performed, and read count-data were obtained from Kim and Kim (2023).

Genes exhibiting a |log_2_FC| > 1 and adjusted *P*-value < 0.05 were identified as DEGs (**Supplementary Table S4**). Functional enrichment analyses based on Gene Ontology (GO) terms and Kyoto Encyclopedia of Genes and Genomes (KEGG) pathways were conducted using the g:Profiler tool (https://biit.cs.ut.ee/gprofiler/). Significantly enriched GO terms and KEGG pathways were identified based on an adjusted *P*-value (FDR) < 0.05.

### Haplotype analysis

A haplotype analysis was performed based on SNPs within regions encompassing the gene-encoding region and the promoter sequence up to 2 kb upstream of the start codon. These SNPs were concatenated and employed to define haplotypes using Haploview version 4.1. Linkage disequilibrium (LD)-based haplotypes were defined using Haploview (Barrett et al., 2005), with haplotype blocks determined by the confidence intervals method (Gabriel et al., 2002). Only haplotypes represented in at least five cultivars were retained for subsequent multiple comparisons. For pairwise comparisons among major haplotypes, Tukey’s honestly significant difference (HSD) tests were applied, with the significance threshold set at 0.05 (**Supplementary Table S6**).

## Results

### Phenotypic variation in salt tolerance across two seedling stages

**Figure 2:**
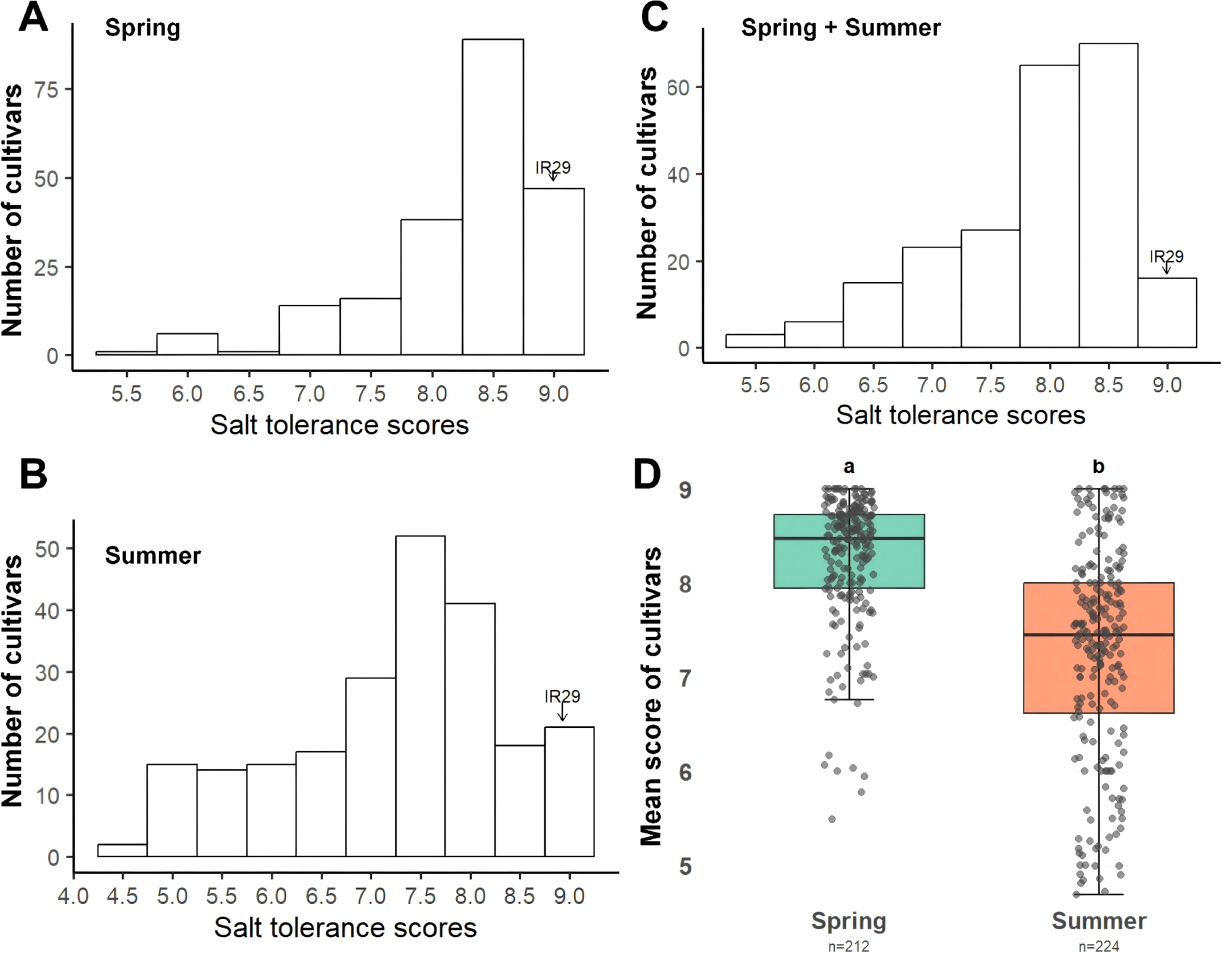
Distribution of seedling salt tolerance scores for 225 rice cultivars across the spring **(A)** and summer **(B)** seasons and the spring and summer seasons combined **(C)**, with the mean cultivar scores of the seasons compared in a boxplot **(D)**.

Seedling-stage salt tolerance was evaluated in 225 rice cultivars across the spring and summer seasons using the modified SES, with lower scores indicating higher tolerance. Across both seasons, the overall mean score was 7.88 ± 1.15 (all ± values are standard deviations), with scores ranging from 5.30 to 9.00, indicating substantial phenotypic variation (**Figure 2C**; **Supplementary Table S2**).

In spring, the mean score was 8.25 ± 1.04, and individual scores exhibited a relatively narrow distribution (**Figure 2A**, **Supplementary Table S2**), whereas in summer the mean decreased to 7.26 ± 0.74, indicating generally improved tolerance at the later growth stage (**Figure 2B**, **Supplementary Table S2**). Among 211 cultivars evaluated in both seasons, 187 showed better performance in summer, 22 performed better in spring, and 2 remained unchanged between the seasons. An overall mean difference of 0.99 ± 0.88 was seen between the seasons (*P* < 0.001), indicating that salt-stress responses were strongly influenced by environmental conditions (**Figure 2D**, **Supplementary Table S2**).

To mitigate environmental biases, we applied a BLUP model that accounted for temporal (season), spatial (block), and genotypic variation, yielding unbiased genotypic estimates for salt tolerance. BLUPs ranged from −2.33 (‘Hanareum4’) to 1.46 (‘Cypress’), with a mean of 0.00 ± 0.92. The cultivars exhibiting the strongest salt tolerance based on the BLUP-adjusted estimates were ‘Hanareum4’, with a combined-season mean of 5.74 ± 1.68, spring mean of 6.00 ± 1.76, summer mean of 4.73 ± 0.70, and BLUP of −2.33; ‘IR73571-3B-11-3-K2’, with a combined-season mean of 5.30 ± 1.63, spring mean of 5.49 ± 1.63, summer mean of 4.69 ± 1.46, and BLUP of −2.24; and ‘Pokkali’, with a (combined-season mean of 5.47 ± 1.03, spring mean of 5.78 ± 1.31, summer mean of 5.20 ± 0.62, and BLUP of −2.07. In contrast, low-salt-tolerance cultivars exhibited much higher tolerance values. For example, ‘Cypress’ exhibited combined-season, spring, and summer means of 9.00, with a BLUP of 1.46 (**Supplementary Table S2**).

BLUP adjustments effectively reduced seasonal bias, as evidenced by their centered distribution and preservation of relative rankings. The five cultivars with the most favorable BLUP-adjusted salt tolerance scores—’Hanareum4’, ‘IR73571-3B-11-3-K2’, ‘Hanareum3’, ‘Geumgang1’, and ‘Pokkali’—each exhibited an overall SES score below 6.00 across both seasons, indicating consistently strong tolerance (**Supplementary Table S2**). These BLUP-adjusted estimates provided reliable genotypic values for subsequent analyses by effectively separating heritable genetic effects from replicate trial variability.

### Genome-wide SNP distributions and data quality assessment

**Figure 3:**
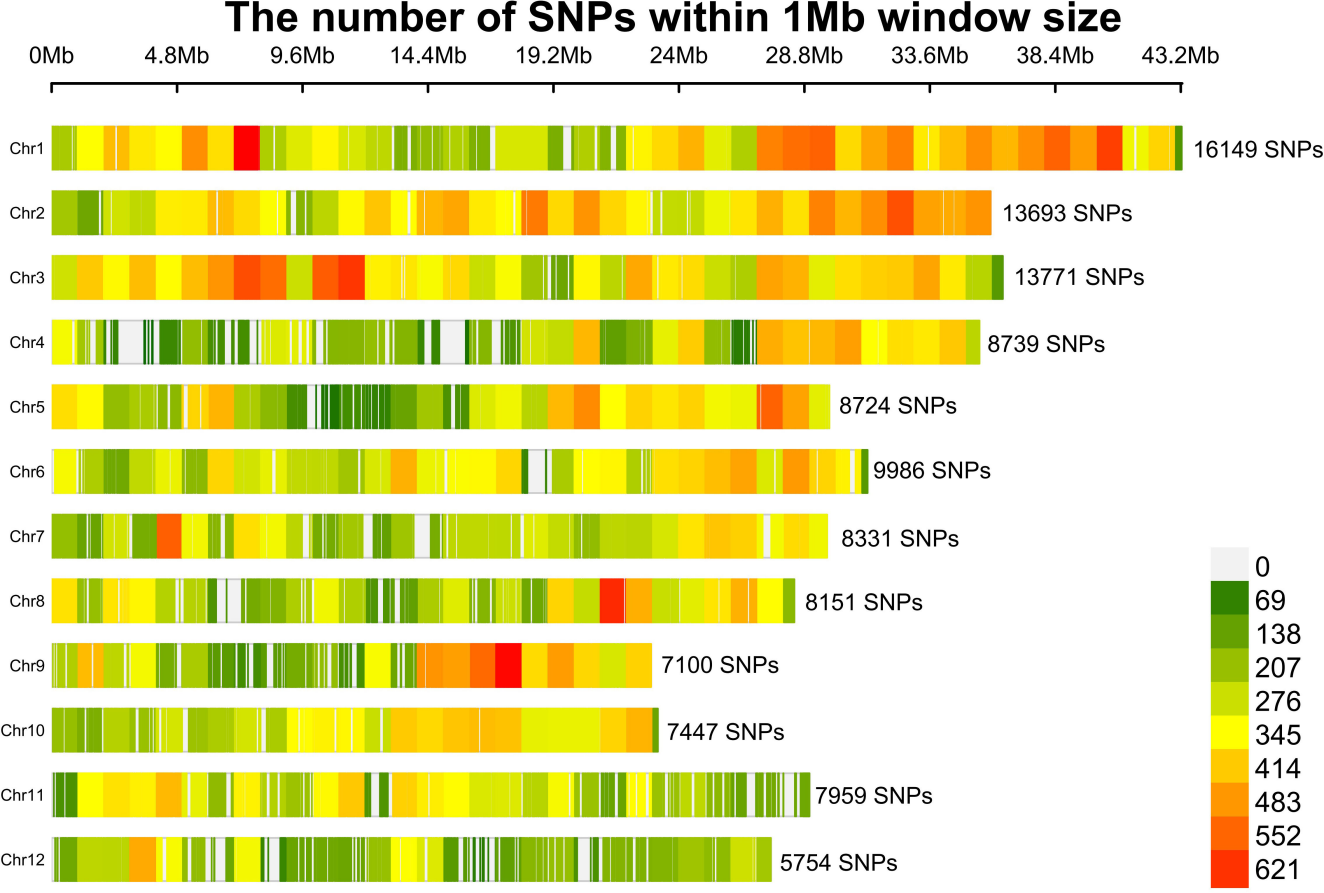
SNP density distributions across the 12 rice chromosomes, based on a 1-MB sliding window.

After filtering SNPs with missing rates > 0.005, a total of 115,804 high-quality SNPs were retained, corresponding to approximately one SNP every 3.5 kb across the 389 Mb IRGSP-1.0 rice genome. Chromosomes 1, 2, and 3 contained the largest proportions of variants, accounting for 14%, 12%, and 12% of the genome-wide total, respectively, whereas chromosome 12 exhibited the lowest variant density (5%), likely due to its extensive centromeric and repetitive regions, which hinder reliable variant calling (**Figure 3**). Empirical studies indicate that a minimum of 40,000 well-distributed SNPs (≈1 per 10 kb) is sufficient to capture most common haplotype blocks in populations comprising a few hundred accessions (Huang et al., 2010; Zhao et al., 2011). Therefore, the current SNP panel provides sufficient genomic resolution to identify causal loci and refine GWAS signals.

### GWAS and QTL identification

A population structure analysis of the 225 rice cultivars identified two subpopulations (*K* = 2, Δ*K* = 2,049) with significant differentiation (*F*_st_ = 0.4249, *P* < 0.001). The STRUCTURE analysis, PCA, and neighbor-joining tree consistently separated the cultivars into *Japonica* and *Indica* groups, and the kinship heatmap showed stronger relatedness within groups than between groups (**Supplementary Figure S1**).

LD decay varied across chromosomes, with chromosome 3 exhibiting the largest half-decay distance (242.31 kb at r² = 0.2) and chromosome 11 the smallest (62.71 kb), as detailed in **Supplementary Figure S2**. Earlier research has shown that LD decay in *Oryza sativa* typically ranges from 100–300 kb, although the extent varies among subspecies and germplasm groups. For example, Mather et al. (2007) reported LD decay of ~200 kb in rice, while Lu et al. (2015) and Huang et al. (2010) detected values between 100–200 kb in diverse landraces.

**Figure 4:**
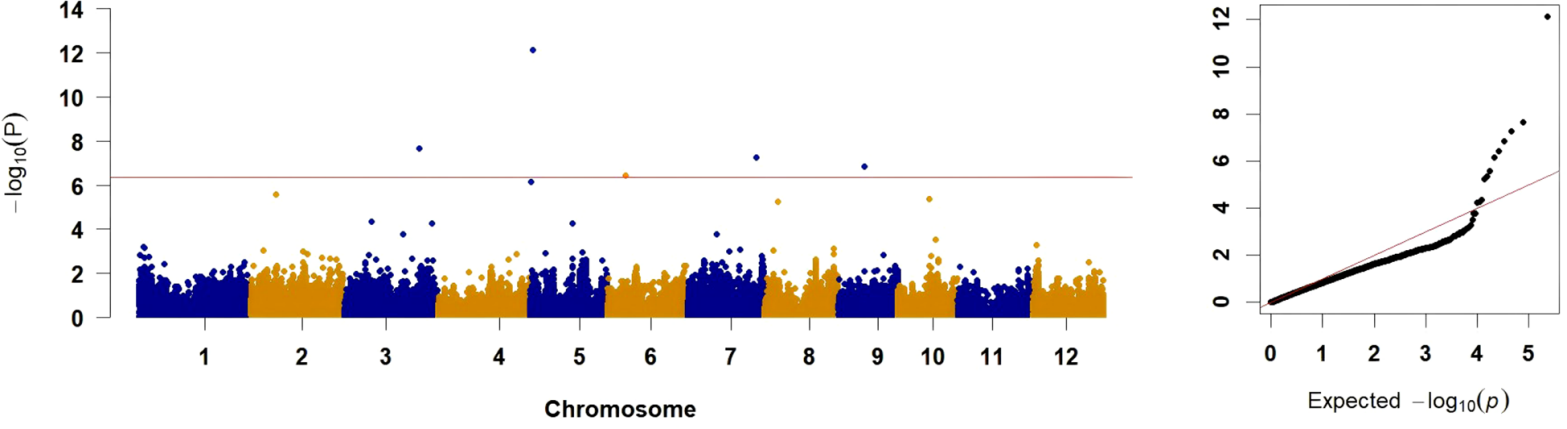
Manhattan and QQ Plots. The left panel shows the Manhattan plot, with a red line at −log10(*P*) = 6.36 indicating the significance threshold. The right panel displays the QQ plot, comparing observed versus expected −log10(*P*) values, with a diagonal red line representing the null hypothesis.

The GWAS was conducted using a FarmCPU model incorporating both fixed and random effects to account for population stratification and kinship. After a genome-wide Bonferroni-corrected significance threshold of −log_10_(*P*) ≥ 6.36 was applied, five significant SNPs, respectively distributed on chromosomes 3, 5, 6, 7, and 9, were identified (**Table 1**, **Figure 4**). The most significant association was for SNP AX-95954242 on chromosome 5 (−log_10_(*P*) = 12.14), whereas SNP AX-154865592 on chromosome 7 explained the largest proportion of the phenotypic variance (22.90%). The remaining SNPs, on chromosomes 3, 6, and 9, exhibited −log_10_(*P*) values of 6.41–7.66, with moderate phenotypic variance explained (0–2.25%). The associated QQ plot confirmed minimal inflation, indicating effective control of population stratification (**Figure 4**).

**Table 1:** Information on the QTLs and associated significant SNPs identified in this study.

QTL	Chromosome	Start position (bp)	End position (bp)	Phenotypic variance explained (%)	Minor allele frequency	Additive effect	Significant SNP	SNP position (bp)	−log_10_(*P*)
*qSES3*	3	29,006,598	29,206,598	2.25	0.10	0.20	AX-115778592	29,106,598	7.66
*qSES5*	5	777,757	977,757	0.04	0.15	−0.32	AX-95954242	877,757	12.14
*qSES6*	6	6,951,455	7,151,455	0	0.05	−0.31	AX-155006483	7,051,455	6.41
*qSES7*	7	26,322,223	26,522,223	22.90	0.26	−0.26	AX-154865592	26,422,223	7.27
*qSES9*	9	10,063,999	10,263,999	0	0.16	0.20	AX-155220002	10,163,999	6.85

Based on a genome-wide linkage disequilibrium (LD) decay rate of 62–240 kb (**Supplementary Figure S2**), a 200-kb interval around each significant SNP was defined as a QTL region. Accordingly, five QTLs, *qSES3*, *qSES5*, *qSES6*, *qSES7*, and *qSES9*, were identified. These QTLs encompassed a total of 134 annotated genes (**Supplementary Table S3**), with individual gene counts of 19 (*qSES3*), 14 (*qSES5*), 21 (*qSES6*), 42 (*qSES7*), and 38 (*qSES9*), respectively.

Among these genes, ten had annotated functions directly linked to salt stress response (**Supplementary Table S3**); these included Os05g0116100 (dehydroascorbate reductase), Os09g0334500 (WRKY transcription factor), Os05g0115800 (MAPK phosphatase), Os03g0717700 (histidine kinase), Os07g0637000 (CBL-interacting protein kinase), Os09g0333500 and Os09g0333600 (ABC transporters), Os06g0237200 (DNA polymerase lambda), Os07g0637400 (Qb-SNARE), and Os03g0719100 (SUMO E3-ligase).

### Salt stress-associated transcriptome profiles in ‘IR73571-3B-11-3-K2’

A transcriptome analysis was performed on the salt-tolerant cultivar ‘IR73571-3B-11-3-K2’ at the seedling stage one day after the initiation of salt stress, with water-treated seedlings serving as the control. We identified DEGS as genes exhibiting a |log_2_FC| > 1 and adjusted *P*-value< 0.05, yielding a total of 834 DEGs, including 140 upregulated and 694 downregulated genes (**Supplementary Table S4**). Among these, prominent gene categories included glutathione S-transferases (Os10g0529800, Os10g0528300, and Os09g0367700), peroxidases (Os01g0294700, Os02g0240300, Os07g0677100, and Os04g0688100), cytochrome P450 (CYP450) family proteins (Os06g0204100, Os10g0525000, Os07g0635500, and Os01g0211600), pathogenesis-related proteins (Os12g0628600, Os12g0555200, Os12g0629700, and Os07g0129200), nucleotide-binding leucine-rich repeat proteins (NB-ARC/NLRs; Os11g0605100, Os08g0174800, Os06g0287700, Os01g0359400, and Os11g0224900), ankyrin repeat-containing proteins (Os09g0343200, Os01g0188900, and Os01g0189100), and various kinases or transporters (Os03g0802500, Os03g0319400, and Os11g0592200).

**Figure 5:**
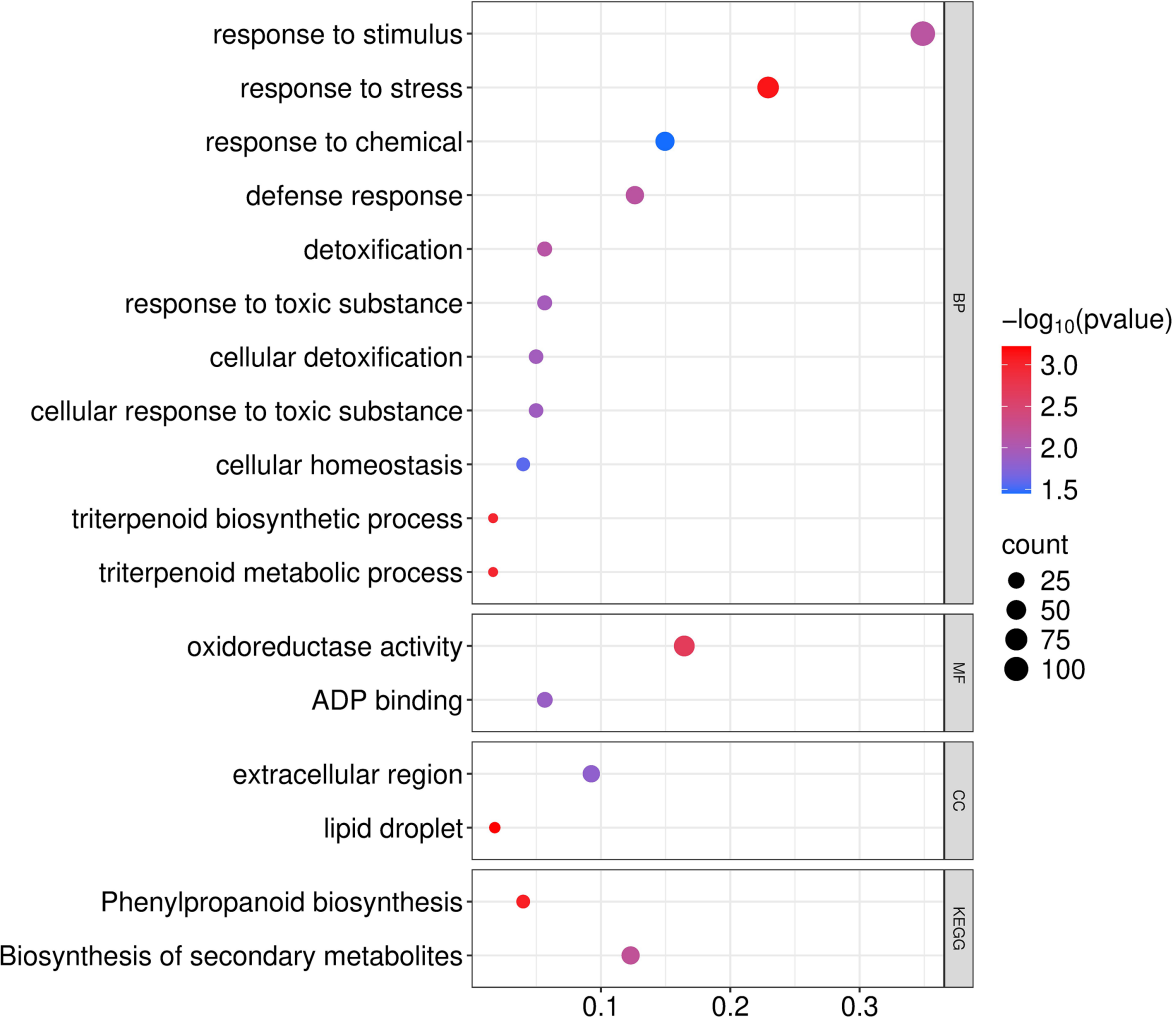
Functional enrichment of DEGs showing significantly enriched GO terms, organized by aspect category (BP, MF, and CC), and KEGG pathways.

We performed functional enrichment analyses for GO terms and KEGG pathways, with significance established at FDR-adjusted *P*-values< 0.05 (**Figure 5**; **Supplementary Table S5**). Among GO terms in the biological processes (BP) aspect category, enriched terms emphasized stress adaptation and protective mechanisms. These terms included “response to stimulus” (105), “response to stress” (69), “response to chemical” (45), “defense response” (38), “detoxification” (17), “response to toxic substance” (17), “cellular detoxification” (15), “cellular response to toxic substance” (15), “cellular homeostasis” (12), and “triterpenoid biosynthetic/metabolic process” (5 each). Significantly enriched GO terms in the molecular functions (MF) category included “oxidoreductase activity” (61) and “ADP binding” (21), while those in the cellular components (CC) category included “lipid droplet” (6) and “extracellular region” (31), implicating redox homeostasis, nucleotide-binding proteins, lipid storage, and extracellular signaling in stress adaptation (**Figure 5**; **Supplementary Table S5**).

The KEGG pathway analysis identified “Phenylpropanoid biosynthesis” (KEGG:00940; 12) and “Biosynthesis of secondary metabolites” (KEGG:01110; 37) as significantly enriched pathways, highlighting enhanced secondary metabolite production for antioxidant defense and structural reinforcement as important functions under salt stress. Intersecting genes in these pathways include peroxidases (Os01g0294700, Os02g0240300, Os07g0677100) and reductases (Os02g0811800) (**Figure 5**; **Supplementary Table S5**).

The integration of the transcriptome data with the GWAS-identified QTLs (**Supplementary Table S2**) identified overlapping genes, such as Os03g0718800 (a protein similar to Physical Impedance Induced protein) in *qSES3*, Os07g0635500 (Cytochrome P450) in *qSES7*, and Os09g0337300 (hypothetical gene) in *qSES9*. The gene Os07g0635500 aligns with the MF-associated GO term “oxidoreductase activity” and KEGG pathway “phenylpropanoid biosynthesis,” suggesting involvement in oxidative stress mitigation and secondary metabolism. Although no direct DEG overlaps were found in *qSES5* or *qSES6*, several QTL-annotated salt-related genes in these QTLs, including Os05g0116100 (dehydroascorbate reductase in *qSES5*), Os07g0637000 (CBL-interacting protein kinase in *qSES7*), Os03g0719100 (SUMO E3-ligase in *qSES3*), and Os06g0237200 (DNA polymerase lambda in *qSES6*), shared functional themes, such as detoxification and homeostasis, with the identified enriched pathways.

### Haplotypes of Os07g0635500

**Figure 6:**
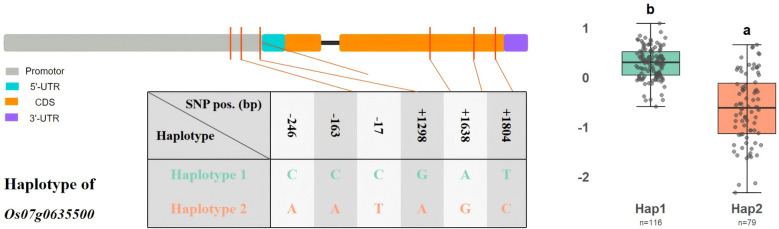
Haplotype structure and phenotypic variation in *Os07g0635500*. The gene model (left) illustrates the promoter (gray), 5’-UTR (cyan), coding DNA sequence (CDS; orange), and 3’-UTR (purple) regions. The SNP positions are marked with red lines. The table shows nucleotide variants for haplotype 1 (Hap1) and haplotype 2 (Hap2), with their positions (in BPs) relative to the beginning of the CDS. The boxplot (right) compares the BLUP salt tolerance scores between the Hap1 (*n* = 116) and Hap2 (*n* = 79) groups, with distinct letters (a, b) indicating significant differences between haplotypes (Tukey’s HSD, *P* < 0.05).

Among the three genes that overlapped between the GWAS-identified QTLs and salt-induced DEGs, Os07g0635500 was prioritized for haplotype analysis. Six SNPs were detected within the 2 kb upstream promoter region and the functional body of gene Os07g0635500 (at positions of −246 bp, −163 bp, −17 bp, +1298 bp, +1638 bp, and +1804 bp, relative to the start codon). Two major haplotypes were defined: haplotype 1 (C-C-C-G-A-T) and haplotype 2 (A-A-T-A-G-C). A comparison of the haplotypes’ BLUP-adjusted seedling-stage salt tolerance scores revealed a significant difference between the haplotypes. Cultivars carrying haplotype 2 exhibited lower BLUP scores than those with haplotype 1, indicating that haplotype 2 confers enhanced salt tolerance (**Figure 6**). A total of 195 rice cultivars were identified, 113 *Japonica* and 3 *Indica* cultivars carried haplotype 1; 46 *Indica*, 30 *Tongil*, and 1 *Japonica* carried haplotype 2 (**Supplementary Table S6**).

### Combined effects of the identified QTLs on salt tolerance

**Figure 7:**
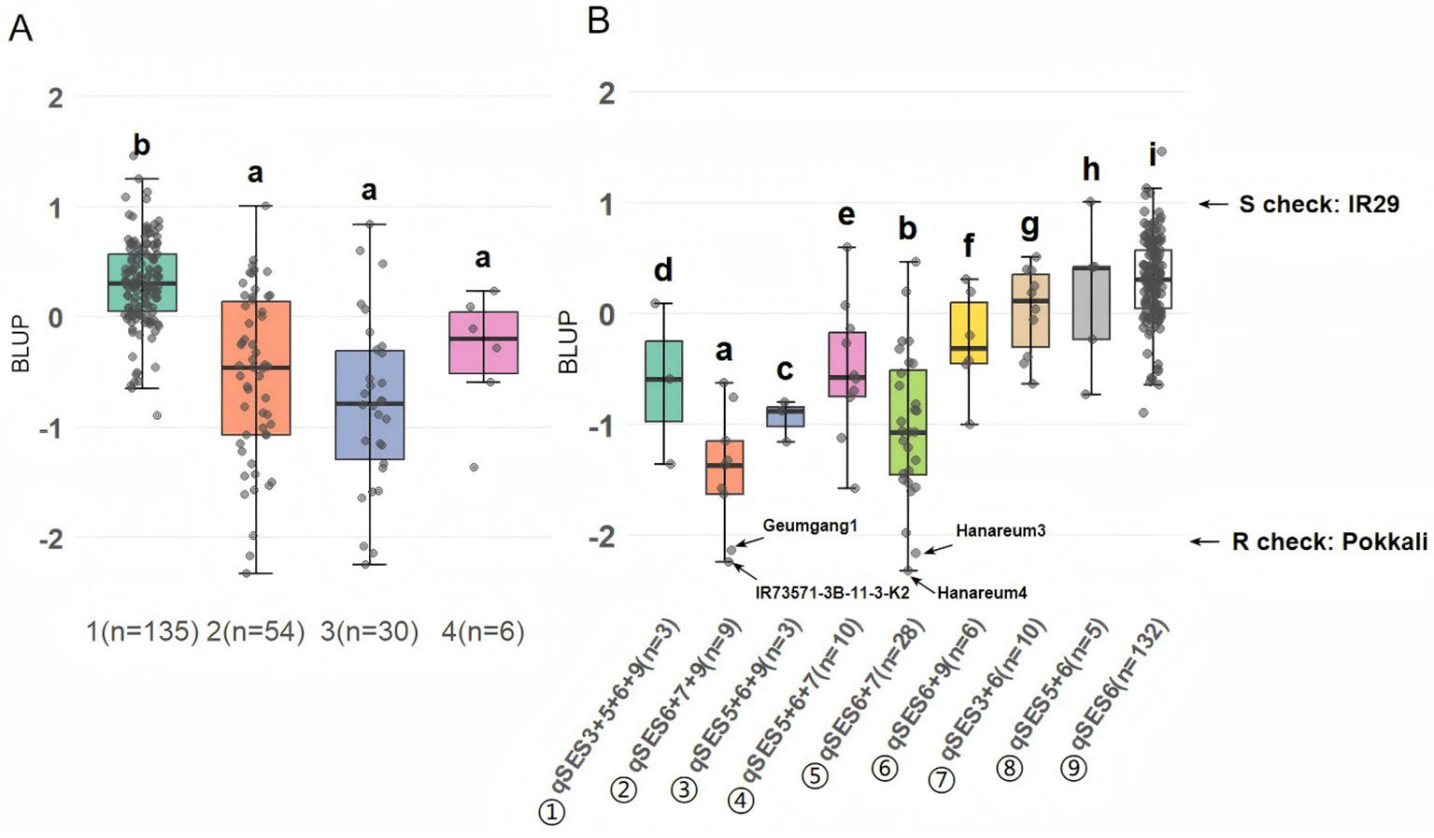
Combined effects of the identified QTLs on salt tolerance. The boxplots show **(A)** the BLUP values of cultivars grouped by the number of favorable QTL alleles they harbor (1–4) and **(B)** the BLUP values of cultivars harboring specific QTL combinations, including only combinations represented by more than three cultivars (*n* ≥ 3). **(B)** includes four outlier cultivars (‘Hanareum4’, ‘IR73571-3B-11-3-K2’, ‘Geumgang1’, and ‘Hanareum3’) showing values lower than those of ‘Pokkali’, a known salt-tolerant cultivar. The letters above the boxes denote significance groupings based on one-way ANOVA followed by Tukey HSD test (*P* < 0.05); cultivar groups associated with different letters had significantly different BLUP scores.

To evaluate the combined effects of the five GWAS-identified QTLs (*qSES3*, *qSES5*, *qSES6*, *qSES7*, and *qSES9*), we grouped 225 rice cultivars by the number of favorable alleles they contained and assessed their BLUP distributions. Among the five identified QTLs, only three were detected individually, each showing relatively high BLUP values, suggesting limited effectiveness when acting alone: *qSES9* (*n* = 1, BLUP = 0.26), *qSES6* (*n* = 132, mean BLUP = 0.30), and *qSES5* (*n* = 2, mean BLUP = 0.72). This shows that QTLs rarely conferred substantial salt tolerance when acting independently. Cultivars carrying two–three QTLs exhibited significantly lower BLUP values than those carrying a single QTL, indicating that synergistic effects dominated. However, cultivars carrying all four QTLs showed higher BLUP values, suggesting that non-linear or epistatic interactions may have reduced tolerance when all favorable alleles were combined (**Figure 7A**; **Supplementary Table S7**).

The combination ⑤ (*qSES6* + *qSES7*), with a mean BLUP of −1.01, appeared to strongly increase salt tolerance, suggesting a strong synergistic interaction. This effect was further enhanced by the additive inclusion of *qSES9* (combination ②), which produced the lowest mean BLUP (−1.43) among the tested combinations. Combination ② can also be compared to combination ⑥, with just *qSES6* and *qSES9*, which exhibited a much higher BLUP of −0.27. Notably, the top four cultivars were associated with these two most-effective combinations: combination ⑤ harbored the highly tolerant cultivars ‘Hanareum4’ (BLUP = −2.32) and ‘Hanareum3’ (BLUP = −2.17), while combination ② contained ‘IR73571-3B-11-3-K2’ (BLUP = −2.24) and ‘Geumgang1’ (BLUP = −2.13). However, the addition of *qSES5* to the *qSES6* + *qSES7* or *qSES6* + *qSES9* combinations (producing combinations ③ and ④, respectively) exerted an antagonistic or epistatic effect, reducing overall tolerance. These patterns highlight *qSES6* and *qSES7* as a core interactive pair, with *qSES9* serving as an additive enhancer and *qSES5* as a potential suppressor (**Figure 7B**; **Supplementary Table S7**).

## Discussion

In this study, we observed substantial phenotypic variation in seedling-stage salt tolerance among 225 rice cultivars. The modified SES scores ranged from 5.30 to 9.00 across both the spring and summer seasons. Several cultivars, including ‘Hanareum4’, ‘IR73571-3B-11-3-K2’, ‘Pokkali’, ‘Geumgang1’, and ‘Hanareum3’, showed superior tolerance (**Supplementary Table S2**). Notably, most top-performing cultivars, including ‘Hanareum4’, ‘IR73571-3B-11-3-K2’, ‘Geumgang1’, and ‘Hanareum3’, belong to the Tongil-type ecotype, which carries a genetic background derived from *Japonica*–*Indica* crosses (**Supplementary Table S1**). This distinction is important for breeding applications, as the introgression of favorable alleles into a pure *Japonica* background may require careful consideration of genetic compatibility and phenotypic stability. These salt-tolerant cultivars represent valuable donor materials for breeding programs, addressing the urgent need for genetic resources to cope with the more than 800 million hectares of salinized agricultural land worldwide (Munns and Tester, 2008).

Significant seasonal differences in SES scores were observed (spring: 8.25; summer: 7.26; *P* < 0.001), indicating that salt tolerance expression was influenced by the environment. One reasonable explanation for this is seasonal variation in the diurnal temperature range: the wider range in spring may disrupt water balance and physiological processes, thereby amplifying salt-induced damage in seedlings (Nikolić et al., 2023). Given that *Japonica* cultivars are typically adapted to temperate climates, their physiological responses to such seasonal fluctuations, such as diurnal temperature range, may differ from those of Tongil-type or *Indica* cultivars. This highlights the importance of G×E interactions when evaluating salt tolerance in *Japonica* breeding programs. Such G×E interactions are common in field phenotyping (Huang et al., 2021; Liu et al., 2019). To reduce environmental confounding and better capture genetic variation, we applied an LMM incorporating season, spatial block, and genotype to generate BLUPs. These BLUP-adjusted scores (mean = 0.00 ± 0.92) centered the distribution, preserved genotype rankings, and corrected seasonal biases, thus providing robust estimates of genetic effects. This multi-environment phenotyping strategy increases the GWAS’s power for detecting salt tolerance loci in rice (Nayyeripasand et al., 2021; Holland and Piepho, 2024).

Our GWAS using BLUP estimates identified five QTLs, *qSES3*, *qSES5*, *qSES6*, *qSES7*, and *qSES9*, on chromosomes 3, 5, 6, 7, and 9, respectively, together harboring 134 annotated genes linked to salt stress responses (**Supplementary Table S3**). These included genes linked to ion transport, redox balance, and signaling. Among the identified QTLs, *qSES3* included the genes encoding SUMO E3-ligase (Os03g0719100; Jmii and Cappadocia, 2021), a protein important for stress conjugation; phytochrome A (Os03g0719800), a protein associated with light signaling; and histidine kinase (Os03g0717700; Sun et al., 2014), a protein involved in cytokinin regulation. The QTL *qSES5* featured genes encoding dehydroascorbate reductase (Os05g0116100; Kim Y. S. et al., 2022), a protein involved in ROS detoxification, and MAPK phosphatase (Os05g0115800; Uchida et al., 2022), which is associated with stress signaling. For *qSES6*, associated genes encoded DNA polymerase lambda (Os06g0237200; Sihi et al., 2015), which aids in DNA repair, and LRR-like protein 1 (Os06g0237502), which maintains chloroplast ROS homeostasis. The QTL *qSES7* harbored genes for Qb-SNARE (Os07g0637400; Zhang et al., 2022), a protein involved in vesicle trafficking, and CBL-interacting kinase (Os07g0637000; Jakada et al., 2025), which is involved in Ca²^+^-mediated Na^+^ exclusion. Lastly, the QTL *qSES9* included genes encoding ABC transporters (Os09g0333500/Os09g0333600; Zhang et al., 2024), which participate in ion efflux, and WRKY transcription factor (Os09g0334500; Li et al., 2025), which is a regulator of stress adaptation. Several favorable alleles identified in this study were found predominantly in Tongil-type cultivars (**Supplementary Table S7**), and their low frequency in *Japonica* backgrounds suggests that marker-assisted introgression strategies will be necessary to incorporate these alleles into elite *Japonica* cultivars without compromising their other agronomic traits.

Transcriptome profiling of the highly tolerant cultivar ‘IR73571-3B-11-3-K2’ revealed 834 DEGs. These DEGs spanned diverse functional annotations, predominantly conserved hypothetical proteins, alongside stress-responsive elements and metabolic regulators. This convergence implies that the DEGs may modulate QTL effects through regulatory networks, highlighting gene candidates for further validation in salt tolerance breeding. Our GO enrichment analysis emphasized processes corresponding with those of the genes located in the GWAS QTLs, including “response to stress,” “detoxification,” and “cellular homeostasis.” For example, the genes Os05g0116100 (dehydroascorbate reductase) and Os03g0719100 (SUMO E3-ligase) are involved in redox balance and protein stability (Kim Y. S. et al., 2022; Jmii and Cappadocia, 2021), while Os09g0334500 (WRKY transcription factor) and Os07g0637000 (CBL-interacting protein kinase) regulate signaling and ion transport (Li et al., 2025; Jakada et al., 2025). The enrichment of genes associated with oxidoreductase activity and ADP binding, as well as KEGG pathways like “phenylpropanoid biosynthesis,” further supports a model in which redox enzymes, transcriptional regulators, and metabolic pathways jointly mediate ROS detoxification, ion homeostasis, and structural reinforcement under salt stress (Munns and Tester, 2008; Pandian et al., 2020; Waseem et al., 2021).

By integrating a GWAS with transcriptome data, we identified candidate genes and regulatory networks underlying salt tolerance (Lv et al., 2022; Zhang et al., 2025; Wei et al., 2024). Three candidate genes were highlighted by the overlap between QTL genes and DEGs: Os03g0718800 in *qSES3* (encoding a physical impedance-induced protein), Os07g0635500 in *qSES7* (cytochrome P450), and Os09g0337300 in *qSES9* (a hypothetical gene). Among these, Os07g0635500 emerged as a prime candidate. The cytochrome P450 play central roles in ROS detoxification, hormone metabolism, and secondary metabolite biosynthesis—processes enriched in our transcriptome data. Under salt stress, Os07g0635500 likely contributes to ROS scavenging and secondary metabolite accumulation, thereby enhancing homeostasis and membrane integrity (Waseem et al., 2021; Nelson and Werck-Reichhart, 2011; Pandian et al., 2020). Haplotype analysis revealed that Haplotype 2 (A-A-T-A-G-C) conferred superior salt tolerance than Haplotype 1, and were predominantly found in Tongil-type and Indica cultivars (**Supplementary Table S6**). The gene Os03g0718800, annotated as encoding a physical impedance-induced protein, may support structural adaptations, such as cell wall reinforcement and osmotic adjustment, which would be consistent with the enrichment in GO terms related to homeostasis and toxic substance responses seen in this study. Although this protein’s precise function remains unclear, related proteins have been implicated in membrane remodeling and ion balance, suggesting protection against Na^+^ toxicity (Colin et al., 2023; Farooq et al., 2021). In contrast, Os09g0337300, a hypothetical gene, while displaying salt-responsive expression, lacks functional characterization.

The additive and subtractive effects of QTL combinations underscore the polygenic nature of complex traits like salt tolerance, where additive and epistatic interactions jointly shape phenotypic outcomes (Bocianowski, 2013). Our analysis revealed strong synergistic effects when two or three QTLs were combined, markedly lowering BLUP values and enhancing salt tolerance, most notably in cultivars carrying *qSES6* + *qSES7* (mean BLUP = −1.01) or *qSES6* + *qSES7* + *qSES9* (mean BLUP = −1.43). Notably, these combinations were prevalent among the elite salt-tolerant cultivars, such as ‘Hanareum4’, ‘Hanareum3’, ‘IR73571-3B-11-3-K2’, and ‘Geumgang1’, all of which exhibited BLUP values below −2.00, well surpassing the population mean.

These findings identify key QTL interactions that can be exploited in marker-assisted selection and genomic breeding to pyramid salt tolerance-conferring genes in rice. In particular, the QTL combination of *qSES6* and *qSES7*, along with the further-enhanced combination containing *qSES9*, represents a promising target for the development of multi-tolerant cultivars. While the combination of *qSES6*, *qSES7*, and *qSES9* showed strong additive effects in Tongil-type cultivars, it remains unclear whether these interactions will confer similar benefits in the *Japonica* cultivars. Future studies should assess epistatic dynamics and phenotypic outcomes following the introgression of the QTLs identified in this study into *Japonica* cultivars.

## Conclusion

This study integrated multi-environment phenotyping, a GWAS, and transcriptomics to elucidate the polygenic basis of seedling-stage salt tolerance in rice. Five QTLs (*qSES3*, *qSES5*, *qSES6*, *qSES7*, and *qSES9*) were identified as harboring stress-responsive genes involved in ion homeostasis, redox balance, and signaling. An analysis of the transcriptome of cultivar ‘IR73571-3B-11-3-K2’ identified 834 DEGs, revealing enriched phenylpropanoid biosynthesis and oxidoreductase activity under salt stress. Through this analysis, the gene Os07g0635500 (*qSES7:CYP450*) was identified as a key candidate for conferring salt tolerance. A synergistic effect was observed in cultivars harboring QTLs *qSES6* and *qSES7*, and salt tolerance was further boosted in cultivars containing these QTLs along with *qSES9*. However, these effects were predominantly enriched in Tongil-type cultivars. These findings provide molecular targets and haplotypes for marker-assisted introgression into *Japonica* cultivars, supporting breeding strategies aimed at improving salt resilience in rice.

## Data Availability

The datasets presented in this study can be found in online repositories. The names of the repository/repositories and accession number(s) can be found in the article/**Supplementary Material**.
