# Supplementary material for: Integrative genomic and transcriptomic dissection of salt tolerance for Japonica rice improvement
Source: Front Plant Sci. 2026 Jan 20;16:1751273. doi: 10.3389/fpls.2025.1751273 (PMC12864118; doi:10.3389/fpls.2025.1751273)
Supplement: Supplementary file 1 [file Image1.pdf]

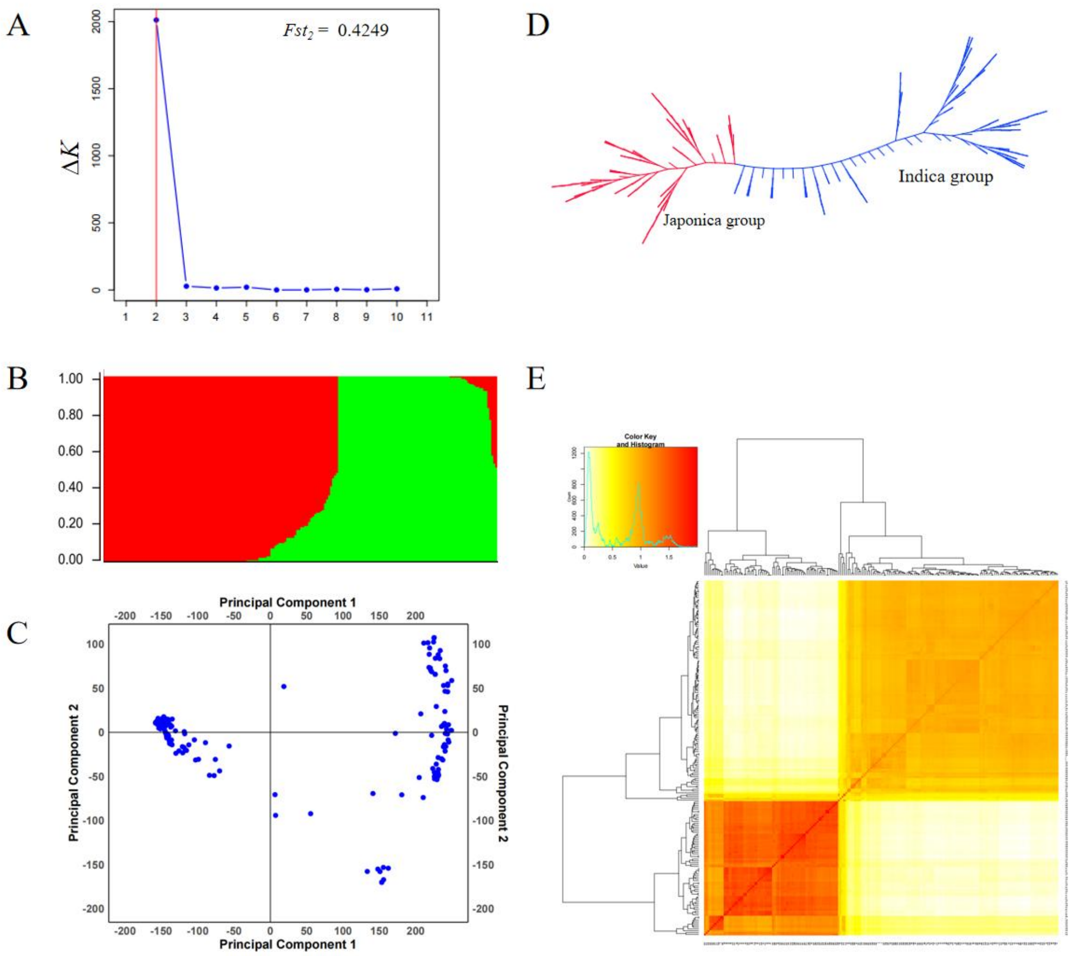

Fig. S1 Population structure of 225 varieties. Panel (**A**) shows the ΔK plot, with a peak at K = 2 (ΔK = 2,049), indicating two subpopulations, and an Fst of 0.4249 ($P < 0.001$). Panel (**B**) presents the STRUCTURE bar plot, with green and red segments representing the two subpopulations across individuals. Panel (**C**) displays the PCA plot, with Principal Components 1 and 2 separating the varieties into two clusters, including outliers. Panel (**D**) depicts the NJ tree, with branches colored blue ('*Indica* group') and red ('*Japonica* group') based on STRUCTURE assignment. Panel (**E**) shows the kinship heatmap, with a color gradient from blue (low relatedness) to red (high relatedness), reflecting pairwise genetic relationships.
