# Supplementary material for: Integrative genomic and transcriptomic dissection of salt tolerance for Japonica rice improvement
Source: Front Plant Sci. 2026 Jan 20;16:1751273. doi: 10.3389/fpls.2025.1751273 (PMC12864118; doi:10.3389/fpls.2025.1751273)
Supplement: Supplementary file 2 [file Image2.pdf]

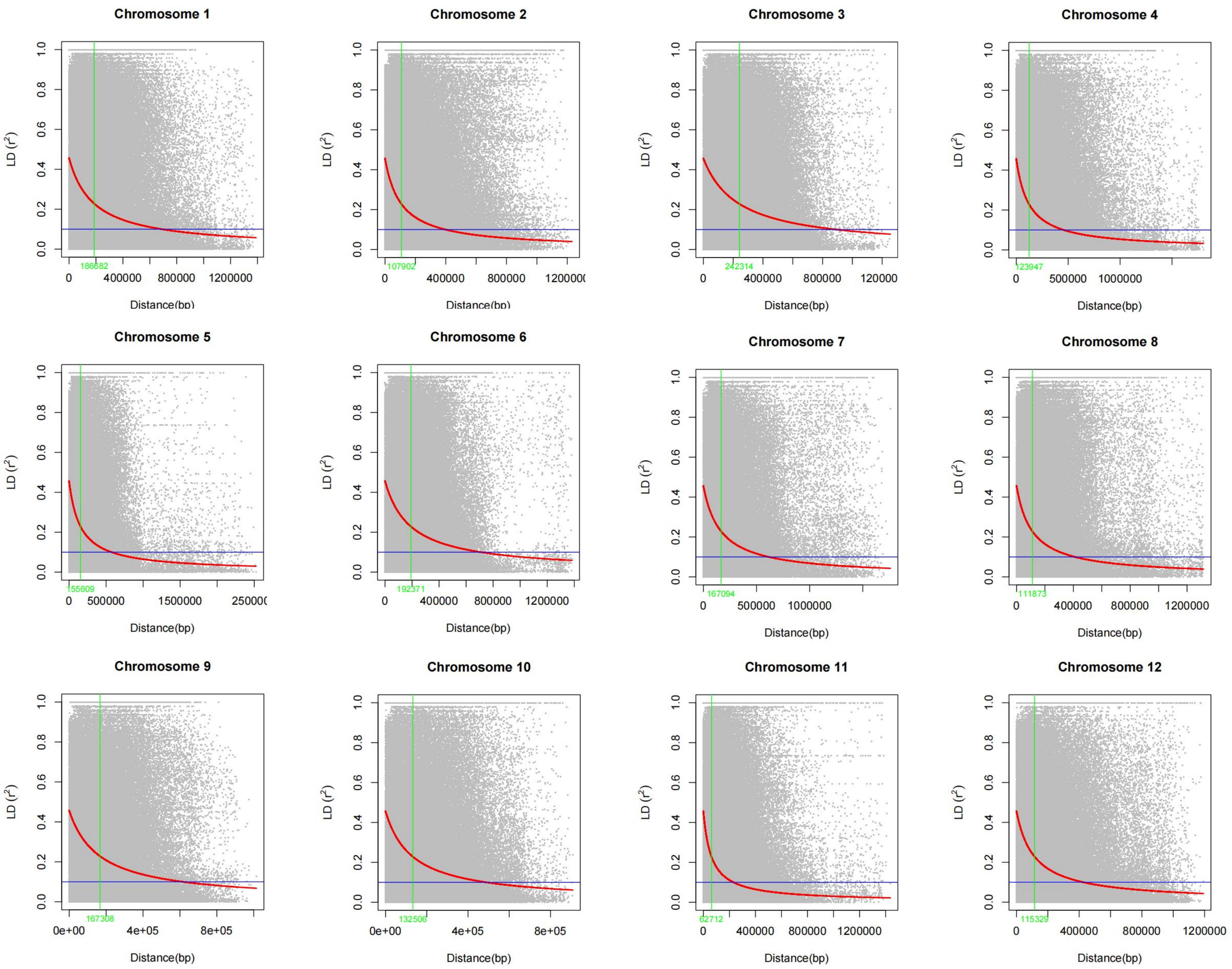

Fig. S2. Genome-wide LD decay patterns across 12 rice chromosomes. Scatter plots show pairwise r^2 values (gray dots) against physical distance (bp). Red lines represent the fitted LD decay curve using a nonlinear regression model. Blue horizontal lines indicate $r^2 = 0.1$, and green vertical lines (where shown) mark the estimated half-decay distance.
